# On the Emergence of Mental Space in Psychology: Interview With Lucas Albert Charles Derks

**DOI:** 10.5964/ejop.v12i2.1186

**Published:** 2016-05-31

**Authors:** Lucas Albert Charles Derks, Alexandru Ioan Manea

**Affiliations:** a Society for Mental Space Psychology, Nijmegen, The Netherlands; b SPER Institute, Bucharest, Romania

**Keywords:** mental space psychology, social panorama, cognitive linguistics, psychotherapy, spatial cognition, cognitive behavioral therapy, spatial representation

## Abstract

In this interview we have the chance to talk with Lucas Albert Charles Derks, founder of the International Laboratory for Mental Space Research and of the Society for Mental Space Psychology and the creator of the Social Panorama approach, about the paradigm that evolved in the last 25 years, entitled Mental Space Psychology, with roots from Cognitive Linguistics, Spatial Cognition and Neuroscience. Today we shall explore the psychotherapeutic approaches which use the Mental Space Psychology, their applicability and their limitations, with a special focus on his own approach, entitled Social Panorama.

**Alexandru Ioan Manea:** In the NLP world, Alfred Korzybski, the founder of “General Semantics”, is famous for his studies in human subjective experience. He used to argue that we perceive reality just through the five senses (sight, hearing, kinesthetic, smell and taste) and upon these we form a map of this reality, thus, never actually operating with the objective reality but just with our perception of it. Ultimately we become the “victim” of our own perceptions and representations, basing upon these the personifications of different events or people with whom we interact. How does the “Mental Space Psychology” relate to the “General Semantics”?

**Lucas Albert Charles Derks:** All these sensory aspects of the model of the world show that they are spatial, that meaning that what we see, hear, feel, taste or smell is connected over space, more exactly that it can be found in a certain area in the mental space. The recognition of this aspect can be found in George Lakoff’s theory from 1987 regarding the spatiality of form. He argued that all the thought process in the human mind is three-dimensional or spatial, and that language is just one-dimensional. So when people communicate, the grammar of language helps them to trans-code from three-dimensional to one-dimensional and back, this meaning that in order to understand what the other person has communicated, the receiver must recreate his own three-dimensional experience. This experience is created from three-dimensional sensory experience into one-dimensional language and back. From my point of view, the essence regarding the work with spatial psychotherapies is that there is no need for a trans-coding of the experience between the psychotherapist and the client, because the psychotherapist does not need to understand his client’s problem, because he can immediately help the client to change his three-dimensional sensory experience. This approach speeds up the process in a considerably way. For example, if a psychotherapist is not using a spatial psychotherapy and is working with a client who is suffering from shyness in contact with a perceived authority, the psychotherapist invites the client to describe the situation in which he is shy and how that significant person is dominating him and the psychotherapist must decode the client’s experience in his own experience. Alternatively, if the psychotherapist is using a spatial-based psychotherapy, he can observe the client describing his own experience and guide him in moving his representations of the person perceived as dominating (downwards and further away), because all these type of images are working efficiently in 99% of authority issues.

**Alexandru Ioan Manea:** After the 1970’s, in psychotherapy began a sort of feud between the experiential-based psychotherapies and the cognitive-based psychotherapies. The “Social Panorama” model that you created seems to have the best aspects of all these sides, the foundation of cognitive linguistics from George Lakoff, Mark Johnson and Gilles Fauconnier, the experiential style of NLP especially, Richard Bandler and the humanistic side of Carl Rogers’s client-centered approach.

**Lucas Albert Charles Derks:** In my opinion, the more a psychotherapist understands the principles of the psychotherapeutic process, the less ritualistic he has to be. In a certain domain like psychotherapy, many practitioners create schemes and algorithms in order to create certainty, but that is just compensating for the lack of insight in the exact principles that he is working with. For example, I will give you a metaphor regarding the repairing of a car, the more you know about the mechanics, the freer you are into repairing it. When psychotherapists are trained, for example, one of the first things they are taught is to make rapport with their client, which is ritualistic. Following the metaphor, in the case of a psychotherapist who has a better understanding about the human mind he can start to intervene right away, because he will be free to do what is best for his client’s problem.

**Alexandru Ioan Manea:** We were talking about psychotherapy, please tell me, in what kind of psychotherapeutic context do you use the “Social Panorama” model?

**Lucas Albert Charles Derks:** I primarily use the “Social Panorama” when clients have difficulties with ongoing relationships, with their families, spouses or children or even at their workplace. I also use it in areas which deal with social personality traits, with habitual patterns regarding how people tend to respond in some situations which end up in conflict, feelings of superiority or of inferiority. The clients don't seem to notice that there is something that can be changed, and they accept that situation as unchangeable, but from my experience these patterns are embedded in early childhood, as most psychologists already discovered. A big part of the social personality is created before age 6 and then this becomes the imprint of the general style of relating to other individuals. For example, with the family members, the person creates a spatial landscape of his relatives in which they have a certain position or role and the person begins to assume that this is her basic role or position in life. Working with clients, I found out that is possible to undo these old roles by going back to the client’s age 5 and exploring how this structure of family was formed and have the clients change it and create a new three-dimensional family image around themselves as if there are at age 5 or so. As they grow up again from that age, the clients can live on in the present and in the future as if they have an honorable position in their family, not needing to stay in the role that they assumed early in life, and they can find a wide range of possible positions or roles on the basis of this new functional family.

[Contrib contnr1]**Alexandru Ioan Manea:** What exactly do you mean by “Spatial Psychotherapies”, how many types of them are there, and which one is suitable for what kind of problem?

**Lucas Albert Charles Derks:** “Spatial Psychotherapies” are approaches in psychotherapy that make use of mental space. For instance, we have “Time Line Therapy”, “Family Constellations”, “Clean Space”, “Social Panorama”, which make use of space in a certain way and in a certain degree. Oddly enough, most psychotherapists in these approaches don't see themselves as much as working with space, because they do not consider it their most important ingredient of their work and they often find other explanations for their results. Their procedures include the spatial manipulations of the mental cognitions of the clients, or orient the clients spatially in a room and move them along locations where there are symbols that mean important concepts to them. Because there are more than just one, but each with its own unique variation, it is more suitable to consider them as one single category, respectively “Spatial Psychotherapies” and to research even further in order to find out what is behind their high impact on the clients. In conclusion, mental space psychology came from the recognition that there is a similar principle behind these therapeutic methods, even if the psychotherapists that use them with their clients do not give mental space that much importance.

“Time-Line Therapy” deals primarily with how people perceive and deal with time. For example, if a client has difficulties to arrive at meetings on time, it may be because he perceives his future as being too far away, or the opposite, if he suffers from severe stress because he perceives his deadlines as being too close. Another issue that can be solved through this approach is regarding the past, where we people store their experiences, memories, traumas, etc... The client can be taken to his past and change past events, or move them so that he can grow from a changed personal history. Also, there are also social differences that can be solved through this approach, because there are cultures where people are more in the here and now, in time, or cultures where people are more in the future and planning ahead. This makes the difference between whether they can or cannot make investments, personal insurance or organizing on the long term.

The “Clean Space” approach works by bringing the clients in new areas of their memory. The client can be helped to break loose from a situation in which they feel stuck by providing them spatial dissociation from that exact location on the floor where they re-experienced that particular and unpleasant feeling. The therapist just keeps them moving to other places, until they find a solution to their problem. This approach can be used for any type of problem because the psychotherapist does not need any kind of content, because he is just simulating the movement. In practice, I saw clients move 4, 5, or 6 times from one location to another in order to solve their issue. Also, it is not suitable for psychotic or depressed clients, because the client needs to be fully concentrated when he makes these kinds of moves.

[Contrib contnr2]The “Family Constellation” approach has two main categories of issues that can be solved. First of all, it is useful in dealing with family issues, because it can help clients to create an alternative view on their family problems. By having an alternative, they can free themselves from issues regarding ideas or feelings towards family members or towards their past. This approach opens up new perspectives because it is often presented as revealing deeper, hidden truths to the client, which has an extra placebo power, because it is a procedure perceived as being somewhat strange, but in the end it has such a high impact on the client, that the final result is taken very seriously by him. Second of all, there are constellation therapies that personify all kinds of abstract concepts coming from the client. For instance, if we take a basic structure, a goal blocked by an emotional problem, the psychotherapist can place people to represent the goal and the emotionality that is in the way of reaching that goal. Now the client can start to (spatially, nonverbally) interact with them, and that may generate more answers than simply communicating about abstract concepts – it may create totally new points of view regarding the issue.

The “Social Panorama” model can be used primarily in dealing with relationships, because the latter are based upon how people represent others and themselves in their mental space. For example, when we talk about self-confidence it’s important to pay attention to how the client perceives his self in space in regards to the others. But when we talk about authority or hatred, it’s more important to pay attention to how others are perceived by the client in his mental space. This approach is also useful in teams or with issues with negative attitudes towards certain groups. For instance, if a refugee comes to a foreign country and he wants to be a part of that society, or culture, he has to perceive his kinesthetic self within his kinesthetic representation of that new group that he wants to belong; he must feel himself in the middle of that group to automatically feel integrated.

**Alexandru Ioan Manea:** You have been using the mental space approach in psychotherapy for nearly 25 years now. What, in your opinion are the limitations of the mental space psychology until now?

**Lucas Albert Charles Derks:** The spatial representations that we work with are probably mostly located in the right brain hemisphere and connected to the basic structures in the hippocampus. These processes are quite in the background; this means they are unconscious processes. In working with clients, they need to become aware of the things that they cannot really be aware of, they need to be open for their intuitive knowledge of were a representation in their mind is located. This is the main limitation, because some clients have difficulties here, they want to have more certainty than they can get from this type of unconscious background knowledge. There are people who are more left brain hemisphere oriented, who don't have too much of this right hemisphere background information present and so they may get stuck when I ask these type of questions, because they want clear facts, things that they can see and not things that they just be vaguely aware of. These are the limitations in my opinion.

**Alexandru Ioan Manea:** Thank you very much Mr. Derks for discussing with you such an important aspect as the new paradigm of “Mental Space Psychology”.

